# *Rhinacanthus nasutus* Improves the Levels of Liver Carbohydrate, Protein, Glycogen, and Liver Markers in Streptozotocin-Induced Diabetic Rats

**DOI:** 10.1155/2013/102901

**Published:** 2013-09-24

**Authors:** Pasupuleti Visweswara Rao, K. Madhavi, M. Dhananjaya Naidu, Siew Hua Gan

**Affiliations:** ^1^Department of Biotechnology, Sri Venkateswara University, Tirupati, Andhra Pradesh 517502, India; ^2^Human Genome Centre, School of Medical Sciences, Universiti Sains Malaysia, Kelantan, 16150 Kubang Kerian, Malaysia; ^3^Department of Biochemistry, Sri Venkateswara Medical College, Tirupati, Andhra Pradesh 517502, India; ^4^Department of Zoology, Yogi Vemana University, Kadapa, Andhra Pradesh 516003, India

## Abstract

The present study was designed to investigate the total carbohydrate, total protein, and glycogen levels in the liver and to measure functional liver markers such as aspartate aminotransferase (AST) and alanine aminotransferase (ALT) in streptozotocin-(STZ-) induced diabetic rats after treatment with methanolic extract of *Rhinacanthus nasutus* (*R. nasutus*). The methanolic extract of *R. nasutus* was orally administered at 200 mg/kg/day while glibenclamide was administered at 50 mg/kg/day. All animals were treated for 30 days before being sacrificed. The amounts of carbohydrate, glycogen, proteins, and liver markers (AST and ALT) were measured in the liver tissue of the experimental animals. The levels of carbohydrate, glycogen, and proteins were significantly reduced in the diabetic rats but were augmented considerably after 30 days of *R. nasutus* treatment. The elevated AST and ALT levels in diabetic rats showed a significant decline after treatment with *R. nasutus* for 30 days. These results show that the administration of *R. nasutus* ameliorates the altered levels of carbohydrate, glycogen, proteins, and AST and ALT observed in diabetic rats and indicate that *R. nasutus* restores overall metabolism and liver function in experimental diabetic rats. In conclusion, the outcomes of the present study support the traditional belief that *R. nasutus* could ameliorate the diabetic state.

## 1. Introduction 

Diabetes is a metabolic disease that is the consequence of a combination of hereditary and environmental factors. This disease causes hyperglycemia and other classical symptoms, especially polyuria, polydipsia, and polyphagia [1]. Diabetes mellitus is a syndrome characterized by the loss of glucose homeostasis as a result of defects in insulin secretion and functionality. The deficiency in insulin causes impaired metabolism of glucose and other energy-yielding fuels such as lipids and proteins [2]. Acute complications include diabetic ketoacidosis, hypoglycemia, hyperosmolar syndrome, and hyperglycemia. Subacute complications include polyuria, lack of energy, thirst, blurred vision, and weight loss. Chronic hyperglycemia leads to the glycation of cellular proteins and may lead to complications affecting the eyes, nerves, kidneys, and arteries [3]. 

Experimental diabetes in animal models has offered extensive insight into the biochemical and physiological alterations of the diabetic state. Many of these modifications, such as changes in the enzymes of glucose and lipid metabolism, have been characterized in hyperglycemic animals [4]. In many cases, structural alterations are oxidative in nature and are linked to the development of vascular disease [5, 6]. In diabetic rats, augmented lipid peroxidation is also linked to hyperlipidemia [7]. Currently, the available therapies for diabetes include insulin and a variety of oral antihypoglycemic agents such as thiazolidinediones, sulfonylureas, and *α*-glucosidase inhibitors. These drugs are used either as a monotherapy or in combination to achieve better glycemic control. All oral hypoglycemic agents are associated with a number of serious, undesirable effects [8]. Plants have played a major role in the introduction of new therapeutic agents [9] and have gained attention as a source of biologically active substances including antioxidants and hypoglycemic and hypolipidemic agents [10–12]. Therefore, scientists have focused on plant sources for new therapeutic agents because plants are natural products and have minimal side effects. For example, the study of *Galega officinalis*, an important medicinal plant, has led to the discovery and synthesis of an important and commonly used antidiabetic drug, metformin [13]. Numerous approaches have been used to investigate the medicinal plants for potential hypoglycemic activities including ethnobotanical survey. The usefulness of plant products is reported to be attributed to the presence of bioactive substances such as flavonoids, alkaloids, essential oils, and phenolic compounds with antioxidant activities [14, 15].

*Rhinacanthus nasutus *(*R. nasutus*) (Linn) belongs to the Acanthaceae family. It has been used to treat numerous diseases such as eczema, herpes, pulmonary tuberculosis, hepatitis, diabetes, hypertension, and different types of skin diseases [16]. In Thai traditional medicine, the root and whole plant of *R. nasutus* have been used for the treatment of *Tinea versicolor*, ringworm, itching, and skin diseases. The leaves have been used for the treatment of fungal infection, skin diseases [17], allergies [18], cancers, and inflammation [19] because *R. nasutus* is a well-known source of flavonoids, steroids, triterpenoids, anthraquinones, lignans, and especially naphthoquinone analogues [20, 21]. Additionally, the root extract of this plant has been used traditionally as an antidote to snake venom [22]. 

Previously, we reported that *R. nasutus* has antimicrobial properties acting against several microorganisms in addition to exhibiting antidiabetic effects [23], hypolipidemic activity, significant *in vitro *and* in vivo* antioxidant activities [24], and amelioration of oxidative enzymes [16]. Insulin regulates the metabolism of many substances by converting the uptake and utilization of glucose in target organs such as liver, skeletal muscle, kidney, and adipose tissue and by controlling the amount of various metabolic enzymes. Because the liver plays a major role in glycogen storage and utilization, a partial or total reduction of the insulin level causes a disruption in carbohydrate metabolism that diminishes the activity of a number of key enzymes, protein, and liver markers [25]. The liver is an insulin-dependent tissue that plays a vital role in glucose and lipid homeostasis and is severely affected in diabetes [26]. The enzymes aspartate aminotransferase (AST) and alanine transaminases (ALT) are two of the key markers for liver function [27]. 

In this study, we investigated the effects of *R. nasutus* on the liver markers AST and ALT and on biochemical parameters (carbohydrate, glycogen, and protein) in experimentally induced diabetic rats to determine if this herb has the potential to be used in the treatment of diabetes. 

## 2. Materials and Methods

### 2.1. Collection of Plant Material

The fresh leaves of *R. nasutus* were collected from Tirumala Hills, Tirupati, Chittoor districts of Andhra Pradesh from July to October 2009. The plant specimen was verified to be the correct species by Dr. Madhava Setty, a botanist from the Department of Botany, S. V. University, Tirupati, India. 

### 2.2. Preparation of the Extract

Fresh leaves of *R. nasutus* (500 g) were shade-dried and milled into fine powder using a mechanical grinder (TTK Prestige, Chennai, India). The powdered plant material was macerated and shaken in methanol using a bath shaker (Thermo Scientific, Mumbai, India) for 48 h. The extract was then filtered with filter paper (Whatman no. 1) and evaporated to dryness under a vacuum with reduced pressure using a rotary evaporator at 40°C. The concentrate was then placed on aluminum foil before freeze drying. The residual extract was dissolved in 1 mL of sterile water before use.

### 2.3. Chemicals

Streptozotocin (STZ) was purchased from Sigma (USA). All other chemicals and reagents used in this study were of analytical grade. Glibenclamide (Sugatrol, Hyderabad, India) was purchased from a local drug store.

### 2.4. Experimental Design

Adult male Wistar rats weighing between 150 and 180 g were obtained from Sri Venkateswara Enterprises, Bangalore, India. They were individually housed in clean, sterile polypropylene cages under standard conditions (12 h light/dark cycles) with free access to standard chow (Hindustan Lever Ltd., Bangalore, India) and water *ad libitum*. Before the commencement of experiments, the animals were allowed to acclimatize to laboratory conditions for one week. The animal experiments were designed and performed in accordance with the ethical standards approved by the local Ministry of Social Justices and Empowerment, Government of India, and the Institutional Animal Ethics Committee Guidelines (Resolution no. 05/(i)/a/CPCSEA/IAEC/SVU/MDN-PVR/dt.13.09.2010).

### 2.5. Induction of Experimental Diabetes

Diabetes was induced by a single intraperitoneal injection of a freshly prepared STZ solution (Sigma, no. 242-646-8) (50 mg/kg in citrate buffer 0.01 M, pH 4.5) to overnight-fasted rats. Diabetes was confirmed by the presence of polydipsia and polyurea as well as by measuring the nonfasting plasma glucose levels 48 h after STZ injection. Only animals that were confirmed to have blood glucose levels greater than 250 mg/dL were included in the study. All the animals were allowed free access to tap water and pellet chow in accordance with the guidelines of the Institute Animal Ethics committee.

The rats were divided into five groups of six animals each as follows.

 Group I: normal rats (controls—animals receiving only buffer).
 Group II: *R. nasutus*-treated normal rats (200 mg/kg/day) [13, 23].
 Group III: diabetic rats (untreated).
 Group IV: *R. nasutus*-treated diabetic rats (200 mg/kg/day).
 Group V: Glibenclamide-treated diabetic rats (50 mg/kg/day).

### 2.6. Acute Toxicity Test

*R. nasutus *(50–250 mg/kg body weight) was orally administered to rats for acute toxicity studies. Each group was observed individually for signs of toxicity and behavioral changes such as hyperactivity, grooming, convulsions, sedation, or hypothermia. These observations began 1 h after dosing and were continued at least once daily for 14 days. The mortality rate was also calculated. 

### 2.7. Biochemical Measurements

At the end of the study (30 days), after an overnight fasting, the animals were sacrificed by cervical dislocation following anesthesia using isoflurane. The liver tissue was excised and washed with ice-cold saline and was immediately immersed in liquid nitrogen and stored at −80°C for further biochemical analysis. Then, the measurements of liver enzyme activity and biochemical assays were performed. 

AST and ALT activities were assayed using the method of Reitman and Frankel [28]. The total carbohydrate content was estimated based on the method established by Carroll et al. [29]. Glycogen content was determined as described by Saifter et al. [30]. The protein content was estimated by the method of Lowry et al. [31] with slight modifications. All enzymatic assays in this study were performed using crude liver homogenate.

### 2.8. Statistical Analysis

The results were expressed as the mean ± SD (*n* = 6). Statistical analysis was performed using one-way analysis of variance (ANOVA) followed by Tukey's test. A *P* value of <0.05 was considered statistically significant.

## 3. Results 

### 3.1. Toxicity Evaluation of Plant Extract

In the acute toxicity study, the methanolic extract of *R. nasutus* did not lead to mortality even at the highest dose of 250 mg/kg body weight in male rats. At the highest dose, no gross behavioral changes were observed among the rats. These results indicate that the toxicity level of *R. nasutus* is low. 

### 3.2. The Effects of *R. nasutus* Extract on Total Carbohydrate Levels in Experimental Rats

**Figure 1:**
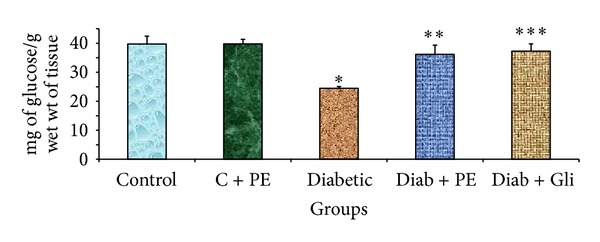
Changes in the total carbohydrate levels in the liver tissue of experimental rats. The bars with different number of asterisks vary significantly at *P* < 0.05. C: control, PE: plant extract, Diab: diabetic, and Gli: glibenclamide.

 The effect of oral administration of *R. nasutus *methanolic extract for 30 days on the total carbohydrate content in liver tissues of control and experimental groups of rats is shown in Figure 1. The liver tissues of diabetic rats showed a significant decrease in the content of total carbohydrate. Treatment with *R. nasutus* methanolic extract or with glibenclamide restored the carbohydrate levels. No significant variations in total carbohydrate levels were found in control rats treated with *R. nasutus* methanolic extract alone.

### 3.3. The Effects of *R. nasutus* Extract on Glycogen Levels in Experimental Rats

**Figure 2:**
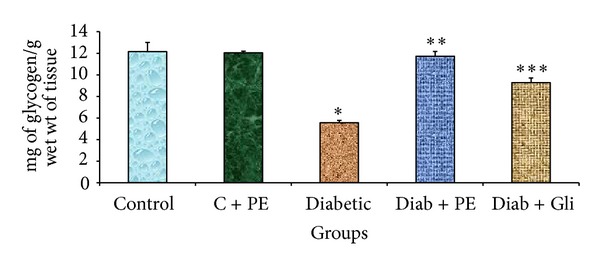
Changes in glycogen levels in the liver tissue of experimental rats. The bars with different number of asterisks vary significantly at *P* < 0.05. C: control, PE: plant extract, Diab: diabetic, and Gli: glibenclamide.

The effects of oral administration of *R. nasutus* methanolic extract for 30 days on glycogen content in liver tissue of control and experimental groups of rats are depicted in Figure 2. The liver tissue of diabetic rats showed a significant decline in glycogen activity. This activity was restored by treatment with *R. nasutus* methanolic extract and by glibenclamide treatment. In contrast, no significant variations were found in the control rats treated with *R. nasutus* methanolic extract alone.

### 3.4. The Effects of *R. nasutus* Extract on Total Protein Levels in Experimental Rats

**Figure 3:**
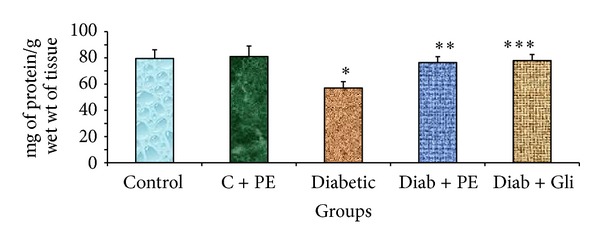
Changes in total protein levels in the liver tissue of experimental rats. The bars with different number of asterisks vary significantly at *P* < 0.05. C: control, PE: plant extract, Diab: diabetic, and Gli: glibenclamide.

The effects of oral administration of *R. nasutus* methanolic extract for 30 days on the content of total proteins in the liver tissue of control and experimental groups of rats are shown in Figure 3. The liver tissue of diabetic rats showed a significant decline in protein content. The amount of protein was restored by treatment with *R. nasutus* methanolic extract as well as by glibenclamide treatment. No significant variations were found in the control rats that were treated with *R*.* nasutus* methanolic extract alone. 

### 3.5. The Effects of *R. nasutus* Extract on AST and ALT Levels of Experimental Rats

**Figure 4:**
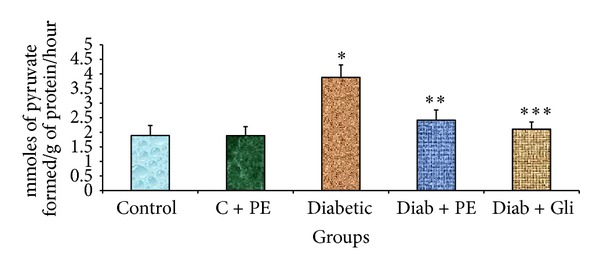
Changes in AST levels in the liver tissue of experimental rats. The bars with different number of asterisks vary significantly at *P* < 0.05. C: control, PE: plant extract, Diab: diabetic, and Gli: glibenclamide.

**Figure 5:**
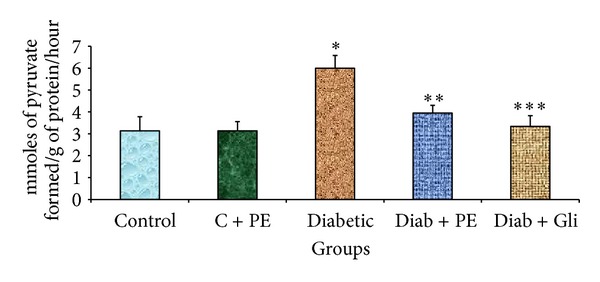
Changes in ALT levels in the liver tissue of experimental rats. The bars with different number of asterisks vary significantly at *P* < 0.05. C: control, PE: plant extract, Diab: diabetic, and Gli: glibenclamide.

The liver tissues of diabetic rats showed elevated AST and ALT levels. The levels of both enzymes were considerably decreased in diabetic rats treated with either *R. nasutus* extract or with glibenclamide. In the control and control treated with plant extract groups, there was no significant change in the AST (Figure 4) and ALT levels (Figure 5).

## 4. Discussion

Plant-derived products usually do not produce any significant side effects when properly administered [32]. Therefore, plants are good candidates for further investigations aimed at increasing the number of the armamentarium against diabetes mellitus. In the present study, STZ was chosen to induce diabetes in rats rather than alloxan. STZ is recognized for its selective destruction to pancreatic *β*-cells [33] and is less toxic than alloxan while maintaining a diabetic condition. It also has an irreversible effect on pancreatic beta cells. Our study is the first to show that *R. nasutus* methanolic extract increases the levels of liver carbohydrate, glycogen, total protein and decreases the levels of liver markers (AST and ALT) in STZ-induced diabetic rats. 

*R. nasutus* methanolic extract significantly increases the total carbohydrate content in the liver tissue of diabetic rats to a similar extent as glibenclamide. Glibenclamide stimulates the insulin secretion from the *β*-cells of the pancreas and is extensively used to treat diabetes mellitus. Glibenclamide mainly acts by inhibiting ATP-sensitive K^+^ (K_ATP_) channels in the plasma membrane [33]. The ATP-sensitive channels inhibition leads to membrane depolarization, activation of voltage-gated Ca^2+^ channels, increased Ca^2+^ influx, and a rise in cytosolic (Ca^2+^) and thereby insulin release. Glibenclamide is extensively used as a standard drug in STZ-induced moderate diabetic model to compare the antidiabetic properties of different types of compounds [34]. The liver is a vital organ that plays an essential role in glycolysis and gluconeogenesis. The organ is the major site for endogenous glucose production [35] producing glucose by either gluconeogenesis or glycogenolysis. Augmented endogenous glucose production, due to either poor pancreatic function and/or reduced glucose clearance, is associated with diabetes and contributes to hyperglycemia [36, 37]. Insulin controls metabolism by regulating the uptake and consumption of glucose in target organs such as liver, skeletal muscle, kidney, and adipose tissue. This regulation is achieved by controlling the actions of various metabolic enzymes. A partial or total reduction of insulin levels will cause a disruption in carbohydrate metabolism, thereby diminishing the activity of a number of key enzymes including glucokinase, phosphofructokinase, and pyruvate kinase [25]. In this study, we observed the ameliorating effects of *R. nasutus* methanolic extract treatment on the carbohydrate content in the livers of STZ-induced diabetic rats. Similar results were shown in the liver, brain and spleen of experimentally induced diabetic rats treated with other herbs such as *Eugenia jambolana *and *Tinospora cordifolia* [32] indicating that *R. nasutus* may have a similar antihyperglycemic effect as these herbs. The increase in total carbohydrate content could be due to: (1) an increase in insulin levels, (2) an enhancement of insulin activity or sensitivity at the peripheral sites, or (3) a reduction of liver gluconeogenesis. Further work is needed to confirm which of these possibilities causes the total carbohydrate content increase. 

Overall, our findings indicate that the use of *R. nasutus* methanolic extract warrants further investigation as an antidiabetic agent or as an adjunct to modern therapies such as glibenclamide. Treatment with *R. nasutus* methanolic extract significantly increased the total glycogen content in the liver tissue of diabetic rats. This effect was similar to the result observed with glibenclamide treatment. The liver preserves normal blood glucose concentrations by storing glucose as glycogen and by generating glucose from glycogen breakdown or from gluconeogenic precursors [38]. Glycogen deposition from glucose is altered in experimentally induced diabetic animals because STZ causes selective damage to pancreatic *β*-cells which results in the decline in insulin levels. The major storage tissues such as liver, kidney, and skeletal muscle depend on insulin for glucose access [39]. Because glucose synthesis in the rat liver is altered during diabetes [40], glycogen content in the liver is also noticeably diminished in diabetes [41]. The liver equilibrates the uptake and storage of glucose via glycogenesis and regulates the release of glucose by activating glycogenolysis and gluconeogenesis [42]. The ability of *R. nasutus* to ameliorate glycogen levels indicates that this plant has the potential to be an antidiabetic agent and should be further investigated.

*R. nasutus* methanolic extract significantly increased the total protein content in the liver tissue of diabetic rats to a similar extent as glibenclamide. The decline in total protein in experimentally induced diabetic rats may be due to microproteinuria, which is a significant systematic indicator of diabetic nephropathy, or to augmented protein catabolism [40, 43, 44]. In diabetic patients with vascular complications, it was also reported that there are major changes in the metabolism of carbohydrates, lipids, and proteins such as augmented lipid peroxidation, dyslipidemia, and other abnormalities [45]. Hyperglycemia is also linked to glucose autooxidation, protein glycation, and the consequent oxidative degradation of glycated proteins that lead to a higher production of reactive oxygen species (ROS) [46]. The increase in total protein content after supplementation with *R. nasutus* indicates that this herb has the potential to lower glucose levels. Similar results were shown in our previous study in which we reported that *R. nasutus *had a significant hypoglycemic effect on STZ-induced diabetic rats and could reduce blood glucose levels [23]. 

*R. nasutus* methanolic extract significantly decreased AST and ALT levels in the liver tissue of diabetic rats to a similar extent as glibenclamide. Any abnormality in or stress on the protein or amino acid metabolism will have consequences in the tissue because these changes drive metabolism towards catabolic products such as ammonia. Free amino acids act as the currency through which protein metabolism operates in the cell [47]. Amino acid metabolism is a complex system involving transamination and oxidation. Transamination is a vital step in amino acid metabolism that involves the transfer of an amino group from one amino acid to the *α*-keto analog of another amino acid. This transfer results in the formation of another amino acid [48, 49]. The enzymes that metabolize the oxidation of the amino acid are known as aminotransferases [50]. Aminotransferase enzymes utilize pyridoxal phosphate, which is a cofactor derived from pyridoxine, as a key component in their catalytic mechanism [51]. Among these enzymes, AST and ALT are extensively distributed in the cells of all animals and provide the link between carbohydrate and protein metabolism by interconverting dynamic substances [52, 53]. AST catalyzes the interconversion of aspartic and *α*-ketoglutaric acids to oxaloacetic and glutamic acids, while ALT catalyzes the interconversion of alanine and *α*-ketoglutaric acid to pyruvic and glutamic acids [54]. These enzymes act as a bridge between protein and carbohydrate metabolisms, and the net result is the inclusion of keto acids into the tricarboxylic acid cycle. The increased levels of AST and ALT activity can be regarded as an indicator for gluconeogenesis [48, 55]. Because *R. nasutus* decreases AST and ALT levels, it is plausible that *R. nasutus *reduces gluconeogenesis and can be further investigated for the treatment of diabetes mellitus.

The dose of 200 mg/kg was selected based on the results of our previous study [23] in which we determined that a 200 mg/kg dose gave a similar effect as a 250 mg/kg dose. The toxicity of the extract was reported in earlier studies and no toxic effects were observed when the herb was orally administered at higher doses. Kupradinun et al. [56] reported that no toxic effect was observed when the extract was used at a dose of 500 mg/kg in animals. Furthermore, *R. nasutus* methanolic extract does not affect the levels of total carbohydrate, glycogen, protein, and liver markers (AST and ALT) in control rats. These findings indicate that *R. nasutus *only affects these markers in diseased conditions and suggests that this herb is safe for consumption by healthy subjects.

Recently, a number of experiments have suggested that medicinal plants have potential roles in ameliorating the effects of hyperglycemia and consequences of diabetes mellitus [40, 42, 57]. Our previous studies also indicated that *R. nasutus* had antihyperglycemic [23] and hypolipidemic effects [58] on STZ-induced diabetic rats. The hypoglycemic effects of *R. nasutus* could be related to its capacity to renovate the damage to liver tissue and scavenge free radicals [16, 24]. In this study, we focused on the effects of *R. nasutus* on the levels of tissue glycogen, carbohydrate, protein, and liver markers such as ALT and AST in STZ-induced diabetic rats. We found that treatment with the herb changes the levels of these markers to be similar to the levels found in nondiabetic rats. Future studies should be conducted to measure the active components of *R. nasutus* extract using gas chromatography or high-performance liquid chromatography to further confirm its potential. The effects of *R. nasutus* extract on pancreas function, especially on insulin production and pancreatic cell rescue should also be investigated. The addition of another group of normal rats administered with glibenclamide will also give further information on the effects of glibenclamide on the STZ group.

## 5. Conclusion

Chronic administration of *R. nasutus* for 30 days resulted in noteworthy improvements in the altered levels of total carbohydrate, total glycogen, total proteins, and AST and ALT activities found in diabetic rats which may contribute to the protective metabolic and hepatic effects of this plant. Overall, our findings indicate that *R. nasutus* may be useful as an antidiabetic drug or as an adjunct to modern antidiabetic therapies such as glibenclamide.
